# Wavefunction realism does not ‘privilege position’

**DOI:** 10.1007/s11229-022-03525-0

**Published:** 2022-02-22

**Authors:** David Schroeren

**Affiliations:** Department of Philosophy, University of Geneva, Rue de Candolle 2, 1211 Genèva 4, Switzerland

**Keywords:** Wavefunction realism, Quantum mechanics, Haecceitism, Ontology, Structuralism

## Abstract

It is common ground among proponents and detractors of wavefunction realism that the view ‘privileges position’, in the sense that it arbitrarily singles out one among a continuum infinity of wavefunction representations as characterizing the fundamental field: the position representation. This paper shows that, properly understood, wavefunction realism does not involve such an arbitrary choice. First, I argue that, though each wavefunction representation gives rise to a different version of wavefunction realism, the difference between these theories amounts to a mere haecceitistic difference. Second, I argue that wavefunction realists should reconceive of their view as a *role-based* thesis that is silent about the relevant haecceitistic differences.

It is common ground among proponents and detractors of wavefunction realism that the view ‘privileges position’, in the following sense. Wavefunction realists claim that there is a fundamental field whose instantaneous distributions are represented by elements of $L^2({\mathbb {R}}^{3n})$, the space of square-integrable complex-valued functions on ${\mathbb {R}}^{3n}$.^1^ But the formalism of quantum mechanics contains a continuum infinity of possible wavefunction formalisms, also known as *wavefunction representations*: one for each complete set of variables of the physical system. Perhaps the most physically salient example is known as the position representation, the formalism in which ${\mathbb {R}}^{3n}$ is interpreted as a fundamental space structurally isomorphic to classical configuration space. Countless other representations are unitarily equivalent to the position representation.^2^ The most well-known example is the momentum representation that is related to the position representation by Fourier transformations. Unitarily equivalent wavefunction representations are physically equivalent, in the sense that they all imply exactly the same empirical predictions.^3^ And yet, wavefunction realists give fundamental ontological significance to the position representation but not to any of the representations to which it is unitarily equivalent: only the position wavefunction is regarded as representing the fundamental field.^4^

It has been argued that this is the source of a potentially fatal problem for wavefunction realism.^5^ According to objectors, the wavefunction realist choice of one among a continuum infinity of unitarily equivalent wavefunction representations is at best arbitrary; indeed, whatever arguments wavefunction realists provide for their view underdetermine the choice between available wavefunction representations. And at worst, wavefunction realism comes down on the wrong side of the unitary equivalence, since it is momentum wavefunctions that are of greater significance in quantum field theory. Call this the *asymmetry objection*.

The goal of this paper is to argue against received wisdom: properly understood, wavefunction realism does not involve an arbitrary choice of the kind that motivates the asymmetry objection. I will focus on the Fourier duality between position and momentum representations; toward the end of the paper, I will explain how our conclusions generalize to arbitrary wavefunction representations.

My argument begins with the observation that the asymmetry objection is a version of a familiar charge against theories that involve haecceitistic commitments. Just like the opponent of spacetime substantivalism complains that the substantivalist posits in-principle-undetectable facts about absolute locations, our objector accuses the wavefunction realist of being committed to in-principle-undetectable facts about which space is inhabited by the fundamental field.^6^ However, I argue that standard moves from that context (in particular, the relationalist and sophisticated substantivalist strategies) face challenges when deployed in response to the asymmetry objection. I ultimately argue that the most attractive strategy for the wavefunction realist is to reconceive of their view as a *role-based* thesis that is not committed to a haecceitistic fact about *which* space is inhabited by the fundamental field, but rather as the thesis that *some* appropriately structured space plays this role.

Here is the plan. Section 1 explains how the Fourier duality suggests two versions of wavefunction realism that are extremely similar to each other, but that nonetheless seem to differ qualitatively. Section 2 argues that the two versions of wavefunction realism (and thus all possible wavefunction representations) in fact differ merely haecceitistically. Section 3 argues that the role-based reconception of wavefunction realism is the most attractive wavefunction-realist response to the asymmetry objection.

## Fourier duality and two kinds of wavefunction realism

Fourier duality suggests that there are two versions of wavefunction realism that are extremely similar to each other.

Recall: according to wavefunction realism, the possible instantaneous distributions of the fundamental field over the fundamental arena are represented by complex-valued square-integrable functions on ${\mathbb {R}}^{3n}$ that form the Hilbert space $L^2({\mathbb {R}}^{3n})$. According to wavefunction realism, the fundamental arena—characterized by ${\mathbb {R}}^{3n}$—is a 3*n*-dimensional space that is the literal container of the fundamental field (and, on certain versions of wavefunction realism, of *all* matter in the universe), in the sense that the fundamental field is an assignment of a property to each point in this space. This space is often called ‘configuration space’ due to its structural similarity to the space whose points each fix a configuration of classical point particles. But it is by now a familiar refrain that the fundamental container of the wavefunction-realist field is not a configuration space in this sense.^7^ According to wavefunction realism, the only distinctive physical role played by the fundamental space is that it is inhabited by the fundamental field. To highlight this feature of the fundamental space, I will henceforth refer to whichever space plays this role as *container space* and wavefunctions on it as *container wavefunctions*.

To understand how Fourier duality suggests an alternative version of wavefunction realism, it will be useful first to review how the duality can be used *within the standard version of the view* to highlight certain physically relevant aspects of the fundamental field. To begin with, each container wavefunction that characterizes some instantaneous distribution of the fundamental field can be written as a superposition

1 $$\begin{aligned} \psi (q)&= \frac{1}{(2\pi )^{3n/2} }\int _{-\infty }^{\infty } \psi (k) e^{-ikq} dk \end{aligned}$$

of plane waves $\psi _{k}(q)=e^{-ikq}$, where *k* designates the wavevector of $\psi _{k}(q)$: a 3*n*-column vector whose direction is the direction of propagation of the plane wave and whose magnitude is its spatial angular frequency—the number of oscillations of the plane wave per unit of space in the direction of propagation, inversely proportional to its wavelength. Within the superposition (1) (also known as the *Fourier decomposition* of $\psi (q)$), $\psi (k)$ is the weight of the plane wave with wavevector *k*. Differently put: $\psi (k)$ is a measure for *how much* of the plane wave with wavevector *k* is present in the Fourier decomposition of $\psi (q)$.

For any instantaneous distribution of the fundamental field, the function $\psi (k)$—which fixes the weights of plane waves in the Fourier decomposition of that distribution—captures substantive physical properties: it captures, for each wavevector, the property that is instantiated by the fundamental field at a time *t* just in case its distribution at *t* (or *t-distribution*, for short) contains a plane wave with that wavevector to thus-and-such a degree in its Fourier decomposition. Henceforth, I refer to the Fourier-dual of container wavefunctions as *wavevector wavefunctions* and to their domain as *wavevector space*. (Note that this space is usually referred to as ‘momentum space’. But this label is inadequate for largely the same reason that ‘configuration space’ is inadequate as a label for container space. Within wavefunction realism, the only distinctive physical role of this space is that it is inhabited by the wavefunction whose value at some wavevector at some instant of time *t* is the weight of the plane wave with that wavevector in the Fourier decomposition of the *t*-distribution of the fundamental field. In particular, just like there are no fundamental entities (such as particles) such that points in container space qualify as their ‘spatial configurations’, there are no fundamental entities such that points in wavevector space qualify as their ‘momentum configurations.’^8^)

Although wavevector wavefunctions capture physical features of the fundamental container-space field, there is a precise sense in which their physical content is already fixed by the container wavefunctions. More precisely, the assignment $\psi (k)$ of weights to wavevectors corresponding to some $\psi (q)$ is completely fixed by $\psi (q)$ through the inverse Fourier transformation

2 $$\begin{aligned} \psi (k)&= \frac{1}{(2\pi )^{3n/2} }\int _{-\infty }^{\infty } \psi (q) e^{ikq} dq. \end{aligned}$$

The relationship between container and wavevector wavefunctions given by (1) and (2) is completely symmetric. Just like (1) is the Fourier decomposition of a container wavefunction into plane waves $\psi _{k}(q)=e^{-ikq}$ on container space, (2) is the Fourier decomposition of a wavevector wavefunction into plane waves $\psi _{q}(k)=e^{ikq}$ on wavevector space. Similarly, just like each container wavefunction fixes the weight of each plane wave in its Fourier decomposition via (2), each wavevector wavefunction fixes the weight of each plane wave in *its* Fourier decomposition via (1).

According to the asymmetry objection, it is just this symmetry between the configuration and wavevector wavefunctions that suggests an alternative to the standard version of wavefunction realism, one which is characterized by the wavefunctions that (within the standard formulation of wavefunction realism) play the role of wavevector wavefunctions. Call the standard version of wavefunction realism just presented *C-wavefunction realism*. It says, of a specific space (call it C), that *it* is the container of the fundamental field. (Think of this as the theory standardly expressed in terms of what we normally refer to as the ‘position’ representation.) The alternative to C-wavefunction realism suggested by Fourier duality is a view I will call *W-wavefunction realism*. It asserts, of a space W distinct from C, that *it* contains the fundamental field. (Think of this as the theory standardly expressed in terms of what we normally refer to as the ‘momentum’ representation.)

For the purposes of the asymmetry objection, the core feature of C- and W-wavefunction realism is that there is a precise sense in which they are Fourier duals of each other. Let a *C-world* be a world at which C-wavefunction realism is true and let a *W-world* be a world at which W-wavefunction realism is true. Moreover, say that a C-world $w_c$ is Fourier dual to a W-world $w_w$ iff (1) at every instant of time *t*, the wavefunctions that characterize the *t*-distributions of the fundamental fields at $w_c$ and $w_w$ are Fourier duals of each other; and (2) so are the laws of dynamical evolution at $w_w$ and $w_c$.

Let me elaborate. Consider a C-world $w_c$ at which the law governing the dynamical evolution of the fundamental field is given by the Hamiltonian

3 $$\begin{aligned} H_c = \left[ - \frac{\hbar ^2}{2m}\frac{\partial ^2}{\partial q^2} + V(q,t)\right] ; \end{aligned}$$

second, a W-world $w_w$ at which the fundamental law governing the dynamical evolution of the fundamental field is given by the Fourier dual of $H_c$

4 $$\begin{aligned} H_w&= \left[ \frac{\hbar ^2 }{2m}k^2 + {\mathcal {F}}[V(q,t)]\right] \nonumber \\&= {\mathcal {F}}[H_c]. \end{aligned}$$

If at every time *t* there is some $\psi (q)$ such that the *t*-distribution of the fundamental field at $w_c$ is characterized by $\psi (q)$ and the *t*-distribution of the fundamental field at $w_w$ is characterized by $\psi (k) = {\mathcal {F}}[\psi (q)]$, then $w_c$ and $w_w$ are Fourier dual. C-wavefunction realism and W-wavefunction realism are thus Fourier duals in the sense that, for every C-world there is a Fourier-dual W-world, and vice versa.

Since the mathematical formalisms of the two versions of wavefunction realism are unitarily equivalent by way of the Fourier transform, they imply exactly the same empirical predictions.^9^ But the similarities between C- and W-wavefunction realism go deeper. To start with, Fourier-dual worlds are amenable to the same mathematical descriptions in terms of Fourier-dual wavefunctions. If $w_c$ and $w_w$ are Fourier dual, then the wavefunctions which at $w_c$ characterize the respective weights of plane waves in the fundamental field characterize the instantaneous distributions of the fundamental field at $w_w$; conversely, the wavefunctions which at $w_w$ characterize the respective weights of plane waves in the fundamental field characterize the instantaneous distributions of the fundamental field at $w_c$. Moreover, there is a sense in which C- and W-wavefunction realism are consistent with the same nomic possibilities. As we just saw, the dynamical law *L* governing the evolution of the fundamental field at some C-world $w_c$ has a Fourier-transform ${\mathcal {F}}[L]$ that governs the evolution of the fundamental field at the W-world Fourier dual to $w_c$. But the scope of ${\mathcal {F}}[L]$ is not limited to the Fourier-dual of $w_c$: at $w_c$, ${\mathcal {F}}[L]$ provides an alternative characterization of the dynamical evolution of the fundamental field by way of determining the dynamical evolution of the wavevector wavefunctions. In this sense, Fourier-dual worlds are models of the same dynamical laws.

Fourier-dual worlds are therefore extremely similar: they agree in their empirical predictions, available mathematical characterizations of the fundamental field, and as regards nomic possibility. Nonetheless, there seem to be significant disagreements. For one thing, the instantaneous distributions of the fundamental field at Fourier-dual worlds generally differ in shape. For example, if the fundamental field in $w_c$ is sharply peaked around some point at some time *t*, then at *t* the fundamental field in a world Fourier dual to $w_c$ will be highly spread out. For another thing, on the assumption that the Schrödinger equations with Hamiltonians (3) and (4) express distinct propositions, the two worlds disagree in their *fundamental* dynamical laws.^10^

This suggests that there are qualitative (though unobservable) differences between C- and W-wavefunction realism. If this were correct, the force of the asymmetry objection would arguably be reduced: it might be easier to put oneself in a state of mind where choices between qualitatively different theories do not seem as objectionably arbitrary as do choices between merely haecceitistically different theories, even if those qualitative differences are in-principle unobservable. However, this point turns out to be moot: as I will now argue, C- and W-wavefunction realism in fact differ merely haecceitistically.

## Merely haecceitistic differences

We can discern some evidence for this claim by considering the modal profile of the fundamental field according to the two theories. In both theories, the possible instantaneous distributions of the fundamental field are characterized by $L^2({\mathbb {R}}^{3n})$. Instantaneous distributions are qualitative: roughly, they concern the shape of the field at an instant—for example, the shape of a Gaussian. So, each instantaneous distribution possible according to one theory has a qualitative duplicate that is possible according to the other theory. Since the total history of the fundamental field can be regarded as a specification, for every instant of time, of an instantaneous distribution of the fundamental field over container space at that time, it follows that the two theories agree about which complete qualitative profiles are possible.

Let me put this point slightly differently. What the above observation suggests is that Fourier duality is not the only relevant relationship between C- and W-worlds. C- and W-worlds can additionally be related by a *permutation* that preserves the qualitative profile of the fundamental field but that changes the fundamental space in which it takes values.

Let us say that two worlds $w_c$, $w_w$ are *permutation dual* iff $w_c$ and $w_w$ agree about everything except that the space that plays the role of container space at $w_c$ plays the role of wavevector space at $w_w$ and the space that plays the role of container space at $w_c$ plays the role of wavevector space at $w_w$. In particular, if the fundamental law at $w_c$ is *L*, then the fundamental law at $w_w$ is the proposition that results from *L* by replacing every occurrence of container space variables in *L* with wavevector space variables, and vice versa. For example, consider a C-world $w_c$ and suppose that at *t* the *t*-distribution of the fundamental field has the shape of a Gaussian. Then the *t*-distribution of the fundamental field at the permutation dual of $w_c$ has the shape of the Gaussian that results from a replacement of configuration variables with wavevector variables. Similarly, if at $w_c$ the dynamical evolution of the fundamental field is given by the Hamiltonian given in equation (3), then at the world permutation dual to $w_c$, the dynamical evolution of the fundamental field is given by the Hamiltonian in which *k* plays the role that *q* plays in (3). As opposed to the Fourier-dual worlds, permutation-dual worlds agree about which fundamental roles are realized; their only difference concerns the haecceistic fact about the intrinsic nature of the spaces that realize those roles.

The permutation dual of a C-world is a W-world, and vice versa. To see this, note that the space inhabited by the fundamental field at a world permutation dual to a C-world is W, and the space inhabited by the fundamental field at a world permutation dual to a W-world is C. Maps that replace C with W (and vice versa) are thus maps between C- and W-worlds.

The upshot of all this is that the difference between C-wavefunction realism and W-wavefunction realism is merely haecceitistic: the two theories disagree merely as regards whether it is C or W that plays the role of containing the fundamental field.^11^

This result generalizes. For each alternative wavefunction representation unitarily equivalent to the standard ‘position’ representation there is an alternative version of C-wavefunction realism that (1) is related to the latter by an analogue of what above we characterized as ‘Fourier duality’ between C- and W-wavefunction realism but that (2) nonetheless differs from C-wavefunction realism merely as regards which space is the container of the fundamental field, and thus differs from C-wavefunction realism merely haecceitistically.^12^

## Role-based wavefunction realism

We can now identify the core of the asymmetry objection. As it is usually understood, wavefunction realism is C-wavefunction realism. As such, it involves a purely haecceitistic commitment to a fact about which space is inhabited by the fundamental field and *a fortiori* involves a choice of one among a continuum infinity of versions of wavefunction realism that differ merely haecceitistically. From this point of view, the asymmetry objection is rather similar to an objection to spacetime substantivalism—roughly, the thesis that there is a manifold of points that are the bearers of geometric qualities. Just like substantivalism is vulnerable to the challenge that it allows for in-principle undetectable differences between possibilities that concern merely which spacetime point plays which qualitative role, wavefunction realism is vulnerable to the complaint that it allows for in-principle undetectable differences between possibilities that concern merely which space is inhabited by the fundamental field.

This suggests a roughly similar landscape of response strategies. First of all, there is the relationalist strategy. The spacetime relationalist denies the existence of the entities about which qualitatively identical substantivalist worlds disagree: spacetime points. Relationalists instead propose a theory that trades exclusively in qualitative geometric relations among concrete entities. The corresponding response to the asymmetry objection is to propose a theory which denies the existence of the kinds of entities about which unitarily equivalent versions of wavefunction realism disagree: the spaces that contain the fundamental field. This response would involve proposing a theory in terms of primitives that correspond to mathematical structure invariant under unitary equivalence: namely, the structure of an abstract Hilbert space with a privileged range of operators on that space. The quantum analogue of the relationalist response would thus result in a type of Hilbert-space fundamentalism, a view that has recently received increasing attention in the literature.^13^

Hilbert-space fundamentalism may be an interesting view in its own right. But it is not a wavefunction realist response to the asymmetry objection. Wavefunction realists are committed to a fundamental ontology of a field on a high-dimensional space; Hilbert-space fundamentalist views are inconsistent with this commitment.

A second possible response strategy mirrors what has become known as *sophisticated substantivalism*. The sophisticated substantivalist agrees with the substantivalist as regards the existence of spacetime points, but denies that permutations of spacetime points result in new metaphysical possibilities. Sophisticated substantivalism is thus the conjunction of substantivalism with a modal constraint along the following lines: if the spacetime points at some world instantiate some qualitative profile, they instantiate this profile at every metaphysical possibility at which they exist. The corresponding response to the asymmetry objection conceives of wavefunction realism as the view that asserts, of some specific space, that *it* is inhabited by the fundamental field, but denies that there is a metaphysical possibility where another space is inhabited by a qualitative duplicate of this field. This sort of ‘sophisticated’ wavefunction realism would block the asymmetry objection by virtue of disallowing the pernicious modal variations in question: according to this sort of view, if C-wavefunction realism is true, W-wavefunction realism is necessarily false; and vice versa.

This response strategy can claim some independent support from certain corners of the fundamentality literature. According to some conceptions of fundamentality, what is fundamental is necessarily fundamental.^14^ On such views, if C is the container of the fundamental field then C is *necessarily* the container of the fundamental field, and so *a fortiori* there is no possibility at which the fundamental field is contained in W.

Nonetheless, those in the grip of the asymmetry objection are unlikely to be satisfied with the sophisticated substantivalist response. The resulting version of wavefunction realism still involves a haecceitistic commitment; the main difference is that this haecceitistic commitment is declared metaphysically necessary. While the view is no longer open to the charge of allowing in-principle undetectable modal variations, the asymmetry objection returns in the guise of a more general worry about underdetermination. Consider two versions of the sophisticated substantivalist response: first, sophisticated C-wavefunction realism, according to which C is necessarily fundamental; and second, sophisticated W-wavefunction realism according to which it is W that is necessarily fundamental. These two theories give inconsistent accounts of the landscape of metaphysical modality: they provide inconsistent accounts of what the metaphysical possibilities are. Modal reality conforms to at most one of the two theories; but there seem to be no principled reasons for deciding which one it is. Differently put: the problem is no longer that our wavefunction realism allows for in-principle undetectable differences between possibilities as regards which space is fundamental; rather, the problem is that our total evidence (including, in particular, arguments for wavefunction realism) seems to underdetermine the choice among a continuum infinity of incompatible ways of imposing the modal constraint that is part of the sophisticated substantivalist strategy.^15^

This suggests that the trouble arising from the asymmetry objection has little to do with whether or not possibilities can differ merely as regards which space contains the fundamental field. Whether there are metaphysical possibilities that differ merely haecceitistically is a question that is likely to turn on fairly general considerations that are not sensitive to local disputes about the ontology of non-relativistic quantum mechanics. Indeed, it would be surprising if it turned out that wavefunction realism would require taking sides in a dispute of this kind.

The trouble for wavefunction realism has its source elsewhere. As we have seen, both proponents and opponents of wavefunction realism have understood the view as the thesis that asserts, of some specific space, that *it* is inhabited by the fundamental field. But this saddles wavefunction realism with a problematic commitment to an in-principle undetectable haecceitistic fact.

This diagnosis suggests that wavefunction realism should be understood as the distinct thesis that *there is some* (appropriately structured) space that is inhabited by the fundamental field: some space, that is, which plays the role of container space. Wavefunction realism, construed as such a role-based thesis, is silent about which space plays this role: the theory is indifferent as regards the intrinsic nature of the space inhabited by the fundamental field. On the role-based conception, wavefunction realism is not the *de re* thesis that asserts, of a particular space, that it is the container of the fundamental field; rather, it is the *de dicto* thesis that there is some space which plays the role of containing the fundamental field.

According to the standard conception, wavefunction realism is C-wavefunction realism and thus involves a commitment to a haecceitistic fact. On the reconception of the view I propose, wavefunction realism does not involve such a commitment: its truth does not hinge on the intrinsic nature of the space that acts as the container of the fundamental field. Granted: on the assumption that there are such things as intrinsic natures, in a world where wavefunction realism is true, there is a haecceitistic fact about which space contains the fundamental field. But this is not because wavefunction realism itself dictates this haecceitistic fact: with regard to the claims that wavefunction realism makes about physical reality, any two appropriately structured spaces are perfectly on a par. On this conception of wavefunction realism, it is not our theorizing that privileges one of these spaces; it is the world that does.^16^

The role-based response to the asymmetry objection is thus distinct from an anti-haecceitist doctrine known as *quantifier generalism*.^17^ According to the latter, fundamental reality is ‘general’ in the sense that no fundamental fact implies that *a particular individual* has some property or stands in some relation; rather, the fundamental facts merely have the corresponding existentially quantified implications to the effect that *there is something* that has this property or that stands in this relation. But role-based wavefunction realism is consistent with the denial of quantifier generalism: it simply has nothing to say about whether reality is ultimately haecceitistic or not.^18^

This illustrates an important respect in which our dialectical situation is different from that of spacetime substantivalism. What is at stake in the debate about the metaphysics of spacetime is precisely whether there are possibilities that differ merely as regards which spacetime points play which qualitative role. In *that* debate, it would not be a dialectical advance to propose a ‘role-based’ version of spacetime substantivalism that merely asserts that *some* spacetime points play the relevant qualitative roles: such a view would simply not be responsive to the central question of the debate. By contrast, wavefunction realism is a response to a certain specific question about the ontology of quantum mechanics: which physical entities (if any) are represented by the wavefunction? It is the central contention of the role-based response to the asymmetry objection that the wavefunction-realist answer to this ontological question does not hinge on whether there are possibilities that differ merely as regards the intrinsic nature of the space inhabited by the fundamental field. This latter question is one on which role-based wavefunction realists are silent.

From a certain perspective, this is as it should be. There is a fairly popular view according to which physics tells us about the fundamental roles that are satisfied in the world rather than about the intrinsic natures of the things that satisfy them. The general idea that physics is silent on the intrinsic natures of the realizers of fundamental roles follows from Lewis-Langton-style ‘humility’ theses according to which, roughly, although such realizers exist fundamentally, the true theory that fixes the intrinsic natures of the actual role-realizers is in-principle unknowable^19^ or even inexpressible.^20^ Another implementation of this idea can be found in the recent structuralist trend in the philosophy of physics according to which only the relevant roles—conceived as ‘structural’ in a way that is cashed out in terms of nomic, causal, geometric or other physically substantive relations such as relations of entanglement—but not their realizers are part of the fundamental ontological inventory of the world.^21^ Both types of approach agree that physics doesn’t tell us about intrinsic natures of the things that satisfy the fundamental roles—either because such natures are in-principle unknowable or inexpressible, or because we have independent reason to think that there are no such things as fundamental role-realizers. Role-based wavefunction realism is consistent with this kind of constraint.

This move also strengthens the adaptability of wavefunction realism to more sophisticated physical theories, such as quantum field theory (QFT). As noted earlier, one aspect of the asymmetry objection is that wavefunction realism comes down on the wrong side of the unitary equivalence between the multitude of wavefunction representations: in QFT, it is the momentum representation (rather than its Fourier dual) that plays a crucial role.^22^ It should be clear that this objection is ineffective against role-based wavefunction realism. This view implies merely that there is *some* appropriately structured fundamental space that contains a fundamental field. Precisely by virtue of its neutrality with regard to the intrinsic nature of the container of the fundamental field, this view can be transposed to settings in which this container has a different structure and plays a different physical role. The move to a role-based conception of wavefunction realism improves its compatibility with future physics.

To be sure: this is not to say that role-based wavefunction realism faces no challenges in the quantum-field-theoretic realm. On the contrary, there is reason to think that these challenges are formidable.^23^ Our result is more limited: the claim that wavefunction realism faces challenges in QFT *as a result of privileging the position representation* is not true.

Our discussion illustrates why the standard conception of wavefunction realism as C-wavefunction realism is a liability for the wavefunction realists’ agenda. One popular argument in favor of wavefunction realism is that it preserves locality and separability (Ney 2021). But such arguments are not sensitive to the differences between C-wavefunction realism and W-wavefunction realism. To begin with, if the fundamental ontology at a C-world is separable, then so is the fundamental ontology at its Fourier dual (and also its permutation dual). Moreover, although Fourier-dual worlds do not agree as regards the locality of the fundamental dynamical laws—as evidenced by the fact that (3) but not (4) gives rise to a partial differential equation with regard to container-space variables—this is not the case for permutation dual worlds: as noted earlier, if the fundamental dynamical law at some C-world is given by (3), the fundamental dynamical law at its permutation-dual world is given by the Hamiltonian in which *k* plays the role that *q* plays in (3). While the (hitherto standard) conception of wavefunction realism as C-wavefunction realism therefore has no bearing on central wavefunction realist arguments, its commitment to a haecceitistic fact about the intrinsic nature of the space inhabited by the fundamental field makes it vulnerable to the asymmetry objection. Our result may thus be put as follows: although proponents of wavefunction realism have undoubtedly conceived of their view as (what I refer to as) C-wavefunction realism, their philosophical ends would have been better served by adopting the role-based conception developed in this paper.^24^

## Conclusion

The starting point of our investigation was the observation that wavefunction realism, as usually introduced, criticized and defended, is vulnerable to what I called the asymmetry objection. We saw that, upon closer inspection, this objection is a species of a familiar kind of objection against theories that involve a commitment to in-principle undetectable haecceitistic facts.

The goal of this paper was to argue that wavefunction realism, understood as a role-based thesis, is not vulnerable to the asymmetry objection. On this view, wavefunction realism is the thesis that implies the existence of a fundamental arena that contains the fundamental field while being silent about the intrinsic nature of this arena.

Some wavefunction realists might protest that something like the role-based conception is what they have always had in mind, and that they were never committed to a claim about the intrinsic nature of the space that contains the fundamental field. However, the fact that prominent wavefunction realists have granted that their view is vulnerable to the asymmetry objection suggests that at least some important implications of a role-based conception of the view have yet to be taken into account. To wavefunction realists who have always tacitly presupposed a role-based conception of the kind elaborated here, I offer this paper as an exploration of some of the hitherto underappreciated strengths of their view.

## Footnotes

1 Another view that has sometimes been referred to as ‘wavefunction realism’ is *multifield realism*: the thesis that the wavefunction represents a fundamental multi-field on three-dimensional Euclidean space—i.e. an assignment of a property to each *n*-tuple of points in this space, where *n* is the number of particles (Hubert and Romano 2018). This view is not the concern of this paper. For an overview of the various types of wavefunction realism, see (Chen 2019).

2 There are at least as many representations as there are canonical transformations of the dynamical variables defining the system. By the Stone-von-Neumann theorem, any two such representations are unitarily equivalent. I thank Wayne Myrvold for helpful correspondence about this point.

3 To be sure: the derivation of such predictions may be more complicated in some representations than in others.

4 That wavefunction realism ‘privileges position’ in this sense is explicit in the original proposals (Albert 1996; Loewer 1996), in more recent defenses and developments of the view (Ney 2012; Ney and Albert 2013; Chen 2017, 2018, 2019; Ney 2019, 2020a, b, 2021) as well as in critical discussions of wavefunction realism (Wallace and Timpson 2010; Myrvold 2015; Dorato 2015; Wallace 2021).

5 In particular, (Wallace and Timpson 2010, p. 706); (Wallace 2021, pp. 65-66); cf. (Ney 2021, p. 137).

6 This argument style is explored in more detail in (Dasgupta 2009, 2016).

7 E.g. (Ney 2020a, p. 4231).

8 This means that wavefunction realists should reject the de Broglie relation $p=\hbar k$, at least insofar as it is understood as a thesis of fundamental ontological significance. To be sure: the *empirical* significance of the de Broglie relation is undeniable: predictions of the diffraction behaviour of electrons based on the de Broglie relation have been confirmed to a staggering accuracy; one notable example is the Davisson-Germer experiment (Davisson and Germer 1927). But it is one thing to accept that a mathematical equation plays an important role in the quantum-mechanical algorithm for deriving empirical predictions; it is another to claim that it is of significance for fundamental ontology. According to the latter ontological conception, the de Broglie relation asserts that the wavevectors of the wavefunction are identical (up to a multiplicative constant) to the momenta of fundamental particles. Clearly, this ontological claim is one that the wavefunction realist must reject: wavevectors are not identical to momenta of any fundamental entities, much less of particles.

9 This is for the familiar reason that the Born rule, the principle by which probabilities are assigned to possible measurement outcomes, is invariant under unitary transformations of wavefunctions.

10 This assumption relies on non-trivial views about fineness of grain: roughly, the Russellian idea that propositions are as fine-grained as the sentences that express them. Straightforward versions of this view are inconsistent due to the Russell-Myhill paradox. Propositional granularity is a deep issue that lies beyond the scope of this paper; (Dorr 2016; Goodman 2017; Bacon 2019). What matters for present purposes is merely that, on the most common coarse-grained accounts of propositions (such as Booleanism or intensionalism, the thesis that necessarily equivalent propositions are identical), the two Schrödinger equations express the same proposition, so that $w_w$ and $w_c$ agree in which propositions are fundamental laws. Thus, if reality is coarse-grained, then Fourier-dual worlds are even more symmetric than they would be on a structured picture.

11 As an anonymous reviewer points out, this use of the notion of a ‘merely haecceitistic difference’ as applying to pairs of worlds that differ merely as regards which space is fundamental is somewhat non-standard. One classic example of merely haecceitistic differences commonly encountered in philosophy of physics concerns possibilities that differ merely as regards which spacetime point plays which qualitative role but that (in particular) agree as regards which manifold of points is spacetime. Although the target of the haecceitistic differences in the present case concerns the fundamental space itself rather than permutations of points in this space, the observation that does the work in both cases is the same: worlds that differ in the relevant respects are qualitative duplicates.

12 An anonymous reviewer raises the question as to whether our argument strategy could be deployed to show that any two theories whose mathematical formulations are unitarily equivalent differ merely haecceitistically. Such an implication would likely be devastating, since it would seem to result in a deeply implausibe deflationism about disputes regarding the ontology of modern physics. However, our argument does not have this implication: unitary equivalence (such as Fourier duality) has implications about the presence of merely haecceitistic differences *only in conjunction with ontological assumptions*—in this case, the thesis that the theories arising from unitarily equivalent wavefunction representations characterize fundamental reality as consisting of a physical field on a high-dimensional space. In particular, this means that our argument does not involve a general claim to the effect that any theory whose mathematical formalism is unitarily equivalent to the mathematical formalism of wavefunction realism agrees with the latter about matters of fundamental ontology. I am grateful to the anonymous reviewer for pressing me on this issue.

13 To my knowledge, (Carroll and Singh 2019) is the first paper-length defense of a Hilbert-space fundamentalism; cf. (Carroll 2021). (Schroeren 2021) contains a detailed development of such a Hilbert-space fundamentalist ontology in the context of non-relativistic quantum mechanics. Some criticisms of views of this kind are articulated in (North 2013) and (Wallace 2021).

14 Cf. (Wildman 2018).

15 In this way, our discussion connects with broader issues concerning the apparent underdetermination of the choice between rival metaphysical packages based on modern physics; see (French and Saatsi 2020) and especially (Callender 2020). I thank an anonymous referee for pointing me to this connection.

16 Note that this conclusion also holds for versions of wavefunction realism that supplement the fundamental field with additional ontological items such as local beables. For example, consider the ‘marvellous point’ version of Bohmian mechanics according to which the fundamental field governs the temporal evolution of a point-like corpuscle in the high-dimensional space. For reasons just presented, this theory does not involve a commitment to the intrinsic nature of the high-dimensional space that contains the fundamental field and the corpuscle; all that it requires is the existence of *some* appropriately structured space that plays this role.

17 (Dasgupta 2009; Dasgupta, 2017; Turner 2017).

18 It is worth noting that, although role-based wavefunction realism is silent about which space plays the role of containing the fundamental field, it *does* require that such spaces are sufficiently structured to be eligible as the fundamental arena of a wavefunction-realist universe. For example, spaces eligible for containing the fundamental field are all amenable to a characterization in terms of ${\mathbb {R}}^{3n}$ and so (among other things) agree as regards their dimension. The haecceitistic differences on which role-based wavefunction realism is silent therefore do not affect the core claim of the wavefunction realist programme: that there is a fundamental field on a (sufficiently structured) high-dimensional space. I thank two anonymous reviewers for pressing me on this issue.

19 (Lewis 2009). (Langton 1998) develops this idea as a reading of Kant’s first *Critique*.

20 (Dasgupta 2015).

21 (Ladyman et al. 2007; Bokulich and Bokulich 2011; French 2014). As an anonymous reviewer points out, structuralist thought has traced important connections between the relationalist and sophisticated substantivalist strategies described above. In a seminal paper on ontic structural realism, James Ladyman (1998) suggests that the Hilbert space fundamentalism—characterized above as the result of applying the relationalist recipe to the multitude of unitarily equivalent wavefunction representations—is a natural consequence of structuralist reasoning about quantum theory. Moreover, it has been argued that sophisticated substantivalism is a form of structuralism (Pooley 2013).

22 (Wallace 2021, pp. 65); (Ney 2020b) and (Ney 2021, Chpt. 4) develop alternative responses to this challenge.

23 Cf. (Wallace 2021; Ney 2021) contains some wavefunction realist responses.

24 I thank an anonymous reviewer for inviting me to elaborate on this issue.
